# Puerperal Eclampsia

**Published:** 1891-03

**Authors:** J. G. Swayne

**Affiliations:** Consulting Obstetric Physician to the Bristol General Hospital and Lecturer on Midwifery at the Bristol Medical School


### PUERPERAL ECLAMPSIA.

BY

J. G. SWAYNE, M.D.,

*Consulting Obstetric Physician to the Bristol General Hospital and Lecturer on Midwifery at the Bristol Medical School.*

In most handbooks on Midwifery, excepting a few of the more modern, puerperal convulsions are divided into three classes; viz., the hysterical, the epileptic, and the apoplectic. It is difficult to understand why such a classification was ever made. There is only one of these three that has any claim to be considered a distinct disease *sui generis;* and that is the epileptic form, or puerperal eclampsia, as it is more correctly termed. The other two are merely attacks of hysteria or apoplexy, which happen to come on during pregnancy or parturition. But, in my own opinion, it would be much more rational to divide cases of puerperal eclampsia into three kinds, according to the time when the attack begins, whether *before*, *during*, or *after* labour; for the prognosis

2

and treatment especially differ widely in each of these varieties. My own statistics of puerperal eclampsia abundantly prove this. Since I have been in practice, during a period of nearly fifty years, I have met with 36 cases of puerperal eclampsia. Of these, 11 came on during pregnancy (before any signs of labour were present), 20 during labour, and 5 after labour. Now, in the eleven that occurred *before* labour, six mothers, or just over one-half, died, and nine children, or more than two-thirds. In the twenty that occurred *during* labour, five mothers, or exactly one-quarter, died, and nine children, or scarcely one-half. In the five that came on *after* labour, no mother and no child died. The prognosis is therefore nearly as favourable as it can be in the third class of cases, and very grave in the first; whilst in the second it occupies an intermediate position.

Before referring more particularly to the prognosis and treatment of puerperal eclampsia, I wish to make a few other passing remarks, from a statistical point of view, on my 36 cases. That 36 cases are a good many to occur, as most of them did, in the private practice of one individual, may be shown by a reference to some very useful statistics, which are drawn from the reports of the Guy's Hospital Charity, and are given in Dr. Galabin's *Manual of Midwifery*. It is there stated that eclampsia occurred once in 842 deliveries; and Dr. Galabin remarks just afterwards, "The general estimate for Europe is about one case in 500 deliveries." With respect to my own experience as to the frequency of eclampsia, I may remark that the only way to form a correct estimate is to carefully eliminate all the cases to which I was called in as consultant, and to record those only in which I was the original attendant. I thus find that there were 5 cases of eclampsia in 1,583 labours, or about 1 in 326, showing that the frequency of eclampsia in my own practice is nearly as much above the average as that of Guy's Hospital Charity is below it. With respect to convulsions in general, whether coming on before, during, or after labour, there are two or three other facts which have been established by others whose observations are still further confirmed by my own: one of these is, the greater frequency of convulsions in first labours; for instance, I find that 23 of my cases were primiparæ, and only 13 multiparæ, the primiparæ being about 66 per cent., whilst in the record of the Guy's Charity they are 60 per cent. I find also that convulsions occur more often in twin cases, as compared with single births. In my 36 cases, there were three of twins, *i.e.* an average of 1 in 12; whereas in ordinary labours twins occur only about once in 80 or 90 cases. Both of these facts would tend to show that the greater tone and tension of the abdominal parietes in primiparæ and the greater distension of the abdomen in twin cases are predisposing causes of eclampsia; in all probability, by the increased pressure thus produced upon the vessels of the kidneys and the consequent derangement of the functions of those organs, which is, with scarcely an exception, the invariable accompaniment of puerperal eclampsia. The honour of discovering the condition of the urine in puerperal eclampsia is due to Dr. Lever of Guy's Hospital, who, in the year 1842, published a paper in the Guy's Hospital Reports to prove that albuminuria is a constant accompaniment of puerperal convulsions. So constant is it, that Dr. Galabin states that "out of all cases in the Guy's Charity during the last forty years, in which the urine was examined, it was free from albumen throughout in only two. The total number of cases in which the presence of albuminuria is recorded is 41." As regards my own cases, I did not make any careful observations as to the state of the urine until 1861; but after that time I did so in every case (with but two exceptions, where I had no opportunity of testing it), and the result was that in all the cases noted, 22 in number, albumen was invariably present.

The prognosis in puerperal eclampsia is always serious. As Dr. Galabin points out, the mortality of the mothers is now reckoned at about 30 per cent., and of the children at about 50 per cent. In my own cases, 11 mothers in 36 died, which is about the same average, and 19 children, which is slightly above this average. All observations, however, tend to show that the earlier the convulsions occur with regard to labour, the more unfavourable is the prognosis. This is shown most conclusively in my own cases, in which, as I have already pointed out, six mothers and nine children died in eleven cases of convulsions *before* labour; five mothers and nine children in twenty cases *during* labour, and none of either in five cases *after* labour. This last is unusually favourable in comparison with the experience of others; for Dr. Galabin states that the maternal loss in those after delivery is on an average about 8 per cent. But these five cases of mine are but a small number from which to draw any very definite conclusions.

I have already pointed out how widely the prognosis of cases of puerperal eclampsia differs according to the time when the attacks came on, whether before, during, or after labour; and I shall now show that there is nearly as wide a difference in the *treatment* of these three classes. This chiefly refers to the question whether it is advisable to hasten delivery. Of course, in the third class, delivery is already accomplished, and is therefore out of the question. In the first class of cases, when there are no signs of labour, it is still a *vexata quæstio;* but I think that in the present day most authorities are agreed that, when all other remedies fail, delivery should be had recourse to as a *dernier ressort.* In the second class of cases, which are by far the most numerous, nearly every authority, I think, is of opinion that it is most desirable to hasten delivery.

There is another point in the treatment of puerperal eclampsia about which medical opinion is not, I think, so unanimous as it ought to be. I refer to the use of blood-letting in such cases. I have long held a very strong opinion as to the value of venesection, and have at various times published cases in support of my opinion. More than twelve years ago an article appeared in the *Medical Circular* for May 15th, 1878, in which the following reference was made to some of these cases: " Dr. Swayne has from time to time brought forward his cases at the Bath and Bristol Branch of the British Medical Association; and in the current number of the *Obstetrical Journal* is recorded one" (No. 28 in my list), "in which the withdrawal of ℥xxx of blood in a full stream from a large orifice was followed by immediate and permanent relief of all the symptoms. The convulsions had gone on for twelve hours before bleeding was resorted to. They had increased in frequency, until at last as many as three took place in an hour, notwithstanding that a full trial had been given to anæsthesia in the form of hydrate of chloral and bromide of potassium; three half-drachms of the former and three drachms of the latter having been given. The bleeding appeared at once to produce a decided constitutional effect. The swollen livid face became pale, the breathing less stertorous, and the pulse soft and feeble, instead of full and throbbing. From that time the fits, which were recurring three times in an hour previously, only returned four times during the next ten hours, and were much mitigated in severity. Signs of labour also came on, and delivery was soon completed with the forceps." I could, if there were time, give many similar cases; suffice it to say, that my own experience has convinced me that in the treatment of puerperal convulsions the three most important remedies are, bleeding, anæsthetics, and delivery. The secondary remedies which were used in most of my cases were purgatives of calomel and jalap, enemata of turpentine and castor oil, and also of assafœtida, cold applications to the head, and counter-irritation by blisters, sinapisms, &c. I need not stop to discuss the efficiency of these last, but will merely state briefly my experience of the first three. On referring to the notes of my 36 cases, I find that in 24 (just two-thirds) bleeding was resorted to, the quantity of blood taken varying from ten to thirty ounces. In 18 it was decidedly beneficial, and appeared to be the most efficacious remedy employed. In nearly every case it was speedily followed by a great diminution in the amount of albumen contained in the urine. To this cause I believe that it owes its great power as a remedy.

By anæsthetics, I mean the inhalation of chloroform or the administration of chloral either by the mouth or rectum. These two remedies, which are nearly similar in their action, are much better than opium, which they have all but superseded. They are both very valuable, especially chloral. I should also mention bromide of potassium, which is an excellent auxiliary. Anæsthetics were employed in 14 of my cases. In four of these the symptoms were much relieved or altogether cut short by chloroform, and in three by chloral. In one case (No. 26) bleeding gave relief for a time; but the fits returning, the patient was kept under the influence of chloroform for seven hours, and had no more convulsions. In another case (No. 22) chloral in large doses was equally efficacious. Delivery, either by the induction of premature labour, by the forceps, by turning or craniotomy, was had recourse to in 14 cases, and in eight gave decided relief. The result of my own experience is, that I am inclined to consider bleeding and anæsthesia as the two important remedies, I can hardly say which is the more important; whilst delivery is little inferior to them, its inferiority being due to the circumstance that it is, of course, not available in *post partum* convulsions, and not always in those that occur before labour. Where it can be applied, the forceps is the best and safest mode of hastening delivery.

As I have frequently referred to my own statistics, and especially to favourable cases which prove the value of venesection, it is but right that I should say something of my unfavourable cases, where venesection and every other remedy failed to avert the fatal termination. They are 11 in all, as follows:

No. 1, Case 9.—Convulsions in seventh month; no sign of labour. Bleeding to 20 ounces, six leeches, cold to head, purgatives, sinapisms, etc. Died undelivered.

No. 2, Case 15.—Convulsions in seventh month. Bleeding to 14 ounces, purgatives, cold to head, chloroform. Fits ceased, but she died soon after giving birth to a dead child.

No. 3, Case 17.—Convulsions at commencement of labour. Seven leeches, chloroform, cold to head, purgatives, etc. Delivery terminated speedily after dilatation with Barnes's bags. No more fits, but she died on the third day from total suppression of urine.

No. 4, Case 18.—Convulsions at full term. Bleeding to 15 ounces, purgatives, cold to head, etc. Gave birth naturally to a dead child. No more fits, but she died comatose the following night.

No. 5, case 20.—Convulsions in eighth month; no sign of labour. Bleeding to 14 ounces, purgatives, cold to head, and blisters. Twenty-six fits before bleeding, and none after. However, she gradually sank, and died undelivered on the next day.

No. 6, Case 23.—Convulsions in eighth month; no sign of labour. Bleeding to 18 ounces, purgatives, cold to head, delivery by turning, chloroform. Fits continued, abating gradually; but she still suffered from some degree of aphasia and dysphagia. She died suddenly of convulsions at the end of three weeks, after eating a hearty meal.

No. 7, Case 25.—Convulsions in ninth month; no sign of labour. Cold to head, purgatives, choral in two doses of 30 grains. No fit for an hour after last dose; os too rigid to turn, delivered by craniotomy; convulsions returned two hours afterwards, and she died.

No. 8, Case 29.—Convulsions in ninth month; no sign of labour. Chloroform, enema of 40 grains of chloral, delivery by forceps, blister to back of neck. Died on third day.

No. 9, Case 31.—Convulsions at full time. V.S. to 10 ounces, tinct. opii. mxv., blister and sinapisms. Died soon after delivery.

No. 10, Case 32.—Convulsions at full term. Bleeding to five ounces, chloroform and an enema of 40 grains of chloral, also an enema of turpentine and castor oil. Died on second day.

No. 11, case 35.—Convulsions at full term. Cold to head and sinapisms, delivered naturally. Fits ceased after labour, and patient went on well, but died at the end of a fortnight from an attack of measles.

I may here remark that these 11 cases refer only to the maternal mortality, and not to that of the infants. There is no need to go into this; the great infantile mortality in puerperal eclampsia is no doubt due to the uræmic condition of the mother's blood, and to the interference with the utero-placental circulation.

It will be observed that six of these cases occurred during pregnancy and before labour—a class of cases which, as I mentioned before, is by far the most dangerous both to mother and child. In four of these cases venesection was not practised, as it was considered to be contra-indicated by the state of the pulse. In some of the others it gave great relief, and in one cut short the convulsions.

In a paper which I published in 1878, and to which I have already alluded, I attributed the efficacy of bleeding in puerperal convulsions to the effect it had of diminishing the amount of albumen contained in the urine. The truth of this opinion was at that time corroborated by the result which had followed the treatment of a number of cases of uræmic convulsions and scarlatinal dropsy by copious venesection. Some of these cases were published by Dr. Robert Kirk in the May and June numbers of the *Glasgow Medical Journal* in 1877, and others by Dr. Bramwell of Perth, in the *Edinburgh Medical Journal* for July, 1875. In every case in which Dr. Kirk tried blood-letting in scarlatinal dropsy the treatment proved eminently successful; whilst in 32 cases of the same disease in which Dr. Bramwell had recourse to general abstraction of blood there was only one death, and that a case which was seen too late for treatment to be of any service. In Dr. West's well-known work, *Diseases of Children*, a similar favourable testimony is given as to the efficacy of blood-letting in such cases.

After all that I have said in praise of bleeding in puerperal eclampsia, I shall no doubt very naturally be asked: How, then, do you account for the very general disuse of this remedy in the present day? I must honestly answer that, as regards puerperal convulsions, I cannot account for it. I know of no good reason which has ever been given against its employment. But yet at the present time this valuable remedy is very seldom used, for no better reason, that I can find, than that in the treatment of all diseases bleeding is quite gone out of fashion. It is true that we have in the latter half of this century acquired two most valuable agents in the treatment of eclampsia—viz., chloroform and choral,—and these have, perhaps, to some extent, caused us to lose sight of the value of bleeding. But in the first half of this century a very different state of things prevailed. Perhaps there was no remedy about which doctors were so thoroughly agreed as about bleeding in puerperal eclampsia. The cautious Denman was not afraid to take away as much as 40 ounces of blood, and Blundell as much as 70. Dr. Ramsbotham, whose book on Midwifery was the usual text-book at that time, says: "Bleeding is our great reliance; the lancet is our sheet-anchor, and blood may be taken to a very great extent: it may be necessary to draw 40, 50, or 60 ounces, nay, even more, in the course of a few hours." Now, however, the case is widely different. Bleeding has been so universally abandoned in the treatment of disease, that the faith of many as to its efficacy in puerperal convulsions has been somewhat rudely shaken. Nay more, there is now such a widespread prejudice against it on the part of the public, that it requires some moral courage on the part of the medical attendant to propose such a remedy, even in the most desperate cases.

All those therefore who, like myself, still believe in it, ought to be ready to give a reason for the faith that is in them. This has been done from time to time by others as well as myself; and I am glad to see that in other countries—in America, for instance—a reaction in favour of this old remedy for puerperal eclampsia is taking place. I lately procured a very useful and sensible little book, by Dr. Landis, an American physician; it is called a *Compend of Obstetrics*, is in the form of question and answer, and is especially adapted to the use of medical students. I cannot do better than conclude by quoting the following advice from it, as to the treatment of puerperal eclampsia:

"What treatment should be employed during the attack?

"1.—Venesection, as the quickest and most powerful means of reducing the vascular tension, cerebral hyperæmia, and secondarily the nervous irritability.

"2.—Chloral and bromide of potassium by the mouth, if the patient can swallow, or in double doses by the rectum.

"3.—Ether or chloroform may be given until the chloral, etc., can be administered and take effect.

"4.—The labour, if in progress, should be terminated as soon as possible without violence.

"5.—Purgatives may be given, especially after delivery.

"What objections are urged against venesection?

"That it is out of fashion, and does not reduce vascular tension in a healthy dog.[1]

"What drug was especially used before the discovery of chloral and the bromides?

"Opium, which relieves the irritability of the nerve-centres.

"What objections exist to its use?

"It allays nerve irritability at the expense of all other indications; when the kidneys are seriously crippled, it may itself cause death; it is no better than other less dangerous remedies."

My own experience, founded on the 36 cases so often referred to, leads me to endorse this opinion; and I may mention that I have recorded that experience by taking copious notes of each case at the time of its occurrence. But as these notes are much too long to be published *in extenso*, all the leading facts contained in them are stated in the synopsis given at pp. 16—21.

I give in detail the record of my thirty-sixth case, because it was one of such unusual gravity that I thought, when I first saw the patient, that it would prove a striking exception to my hitherto favourable experience of *post partum* eclampsia. It is as follows:

On August 20th, 1890, I was summoned to Weston-super-Mare to see Mrs. H.—a primipara, æt. 37—in consultation with Mr. Rossiter and Dr. Fraser. I arrived about 4 p.m., August 20th, and was told that she was confined about 7 a.m. on the previous day. The labour was rapid and in every respect favourable. The child

[1] Dr. Landis, however, adds immediately afterwards: "Veratrum viride may be used instead, if there is time to wait upon its action." This remedy is at present much praised in America. I have not yet tried it, but ntend to do so at the first favourable opportunity.

was born alive, but was rather small. She went on well until 9 a.m. on the next day, when she was suddenly seized with a violent epileptiform convulsion. She did not regain consciousness after the fit, but remained insensible with dilated pupils and, occasionally, stertorous breathing.

Mr. Rossiter, who was the original attendant, found that there was albumen in the urine, the deposit forming about one-fifth of the whole. Having sent for Dr. Fraser, they put some leeches on her temples and cupped her over the kidneys; but Mr. Rossiter did not think that the amount of blood thus obtained exceeded ℥iij. They also gave her ʒi. of potassic bromide in four doses, a purge of calomel and jalap, and one drop of croton oil. Yet, notwithstanding these measures, the fits recurred—at first about one every hour, and then more frequently.

I arrived about 4 p.m., and she then had another fit, being the twelfth. After a consultation, I bled her to ℥xvj. The pulse, which before this was hard and bounding, became softer and quicker, and the countenance, which before was very flushed, became paler after the bleeding; and she began to show some signs of sensibility, and tried to get out of bed to have a motion, as her bowels began to act freely. However, about a quarter past five she had another fit, but not so severe as the last; and, after a longer interval, we ordered her 15 grains of potass. bromide and 20 grains of chloral hydrat., to be given by enema in half-an-hour's time, and to be repeated if necessary. I then had to leave to catch the Bristol train.

I did not hear anything more about her until August 26th, six days after this, when I received a letter from Mr. Rossiter, with the following account of her progress:

"For the next five hours after your visit the symptoms underwent little change; but the attacks were rather less severe and at rather longer intervals. After 10 p.m., however, copious purging commenced, and the convulsions did not recur. Consciousness did not return until the afternoon of the next day. I need hardly add, the Wednesday and part of Thursday remain a complete blank. Since then, the breasts have become slightly enlarged, but are now subsiding. The lochia have been very scanty and irregular. The temperature ranges from normal to 100°. But unfortunately the amount of albumen is on the increase to-night—about one-half. I am dry-cupping the loins, purging moderately, and giving mild diaphoretics."

I did not hear from Mr. Rossiter again until September 19th (exactly a month after delivery). In his letter he told me that Mrs. H. was then "quite convalescent, coming down to breakfast and driving about daily." He also added: "Since I last reported the albuminuria has steadily diminished from one-half to a decided trace only. There has been a great deal of pus and mucus, and some blood, but no discoverable casts; and the occurrence of vesical irritation with it, makes me think that a good deal of this was from cystitis. During the whole of the second week the temperature, which had previously been nearly normal, ranged from 101° to 102°, possibly from the above cause."

On reviewing the history of this case, it will be observed that the first fit came on the day after delivery, that it was a violent one, and that after it the patient lay in an unconscious state with dilated pupils and stertorous breathing. Some leeches were applied to the temples, and by this means about ℥iij. of blood were obtained. ʒi. of bromide of potassium was given in four doses, as well as a dose of calomel and jalap, and one drop of croton oil. Yet, notwithstanding these measures, the fits recurred—at first every hour, and then more frequently. I arrived about 4 p.m., and, after a consultation with the medical men in attendance, bled her to ℥xvj. The pulse, which before this was hard and bounding, became softer and quicker, and the countenance, which was before very flushed, became paler; and she began to show slight signs of consciousness. She remained in this condition for about five hours; but the attacks were less severe and at longer intervals until about 10 p.m., when copious purging commenced, and after this the convulsions did not recur; but yet complete consciousness did not return until the next day. From all this it appears that the improvement in the symptoms commenced immediately after the bleeding, and was completed by the copious purging which subsequently ensued. This case is only one out of many others which I have published from time to time, in order to prove the efficacy of venesection in puerperal convulsions, and thus to rescue, if possible, from unmerited oblivion a most valuable but, in the present day, most unaccountably neglected remedy.

---

NOTE.—Since writing this paper my attention has been called to an article in the *British Medical Journal*, January 31st, 1891, called the "Revival of Venesection," referring to a valuable paper by Dr. Pye-Smith of Guy's Hospital, which was discussed at the last meeting of the Royal Medical and Chirurgical Society of London. Dr. Pye-Smith's conclusions (which were very favourable to venesection) were based on a record of fifty cases of various diseases in which bleeding had been resorted to with advantage. It is, therefore, very gratifying to find that I am not alone in my attempts to rescue so important a remedy from oblivion.

| No. | Name and Residence. | Date of Attack. | Original Attendant. | Consultant. | Whether fits came on before, during or after labour. | Whether Multipara or Primipara. | Presentation. |
|---|---|---|---|---|---|---|---|
| 1 | Sarah Higgs, Eugene Street | 1841. | Dr. Swayne (when a pupil) | Mr. Swayne, Sen., Lecturer on Midwifery | During | Multip. | Vertex |
| 2 | Mary Tilman, Albion Row, Bedminster | 1842. | Do. | Do. | During | Primip. | Feet |
| 3 | Not stated | 1843. | Mr. Bridges (a pupil) | Dr. Swayne | During | Multip. | Twins—Both vertex |
| 4 | Mrs. Leaver, Hotwells | 1847. | Mr. Wheeler | Dr. Swayne | During | Primip. æt. 30. | Vertex |
| 5 | Not stated | 1849. | Mr. Taylor (pupil) | Dr. Swayne Lecturer on Midwifery | After | Primip. | Vertex |
| 6 | Mrs. Hodge, Jacob's Wells | 1849. | Dr. Swayne | None | During | Multip. | Vertex |
| 7 | Mrs. Jefferies, Stokes Croft | 1850. | Dr. Herapath | Dr. Swayne | During | Primip. | Vertex |
| 8 | Mrs. G., Somerset Sq. | 1853. | Mr. Godfrey | Dr. Swayne | During | Primip. | Vertex |
| 9 | Martha M., Cox's Buildings, St. Philip's | June 28th, 1854. | Mr. Corbould | Dr. Swayne | Before In 8th Month | Primip. | No dilatation of os uteri |
| 10 | Jane Fussell, Temple Street | Mar. 13th, 1856. | Mr. Biggs (pupil) | Dr. Swayne | During | Primip. æt. 19. | Vertex |
| 11 | Mrs. — Old Market St. | Dec. 4th, 1856. | Mr. Coe | Dr. Swayne | During | Primip. | Vertex |
| 12 | Mrs. B., Buckingham Vale | Sept. 4th, 1861. | Mr. Parker | Dr. Swayne | During | Primip. æt. 30. | Vertex |

| No. | Result to Mother. | Result to Child. | State of Urine. | Period of Attack and Duration of Convulsions. | Treatment, &c. |
|---|---|---|---|---|---|
| 1 | Recovered | Lived | Not stated | Convulsions came on with the last pains; ceased within a fortnight. | Venesection to ounces xx. Head shaved, and cold applied; Enemata of turpentine, castor oil and assafœtida. |
| 2 | Recovered | Died convulsed after 2 hours | Do. | The fits came on when the os uteri was the size of a shilling. | V.S. to ounces xxx. Only two fits after venesection. Purgatives and antispasmodics given. Delivery hastened. |
| 3 | Recovered | Lived<br>Lived | Do. | Fits came on just before delivery, and ceased within 24 hours. | *Post partum* hemorrhage, and therefore no V.S. Purgatives and antispasmodics given. |
| 4 | Recovered | Dead (putrid) | Do. | Convulsions lasted 12 hours; ceased after delivery. | V.S. to ounces xx. Croton oil 1 drop, calomel gr. x. (before I arrived). Enema terebinth. and ol. ricini. Delivery by short forceps. |
| 5 | Recovered | Lived | Do. | Convulsions came on 6 hours after delivery; lasted some hours. | V.S. to ounces xxiv. Calomel and jalap; castor oil and turpentine; cold to head; enema and antispasmodics. |
| 6 | Recovered | Lived | Do. | Convulsions came on during first stage; nearly ceased in second. Two or three fits after labour. | No V.S. on account of her low, bloodless condition. Ammonia and antispasmodics given. |
| 7 | Recovered | Dead | Do. | Fits came on early in first stage, with complete insensibility between. | V.S. to ounces xij. Purgatives. Delivery by craniotomy, because os uteri was not dilated enough for forceps. |
| 8 | Recovered | Lived | Do. | Fits came on late in second stage; ceased after delivery. | V.S. to ounces xx. Short forceps used. |
| 9 | Died (No P.M.E.) | Died | Do. | No sign of labour; convulsions lasted 19 hours. | V.S. to ounces xx. Twelve leeches to temples; cold to head; enema of turpentine and castor oil; 1 drop of croton oil. |
| 10 | Recovered | Dead | Do. | Convulsions came on near end of labour, and lasted more than 24 hours. | V.S. to ounces xvj. Head shaved; cold applied. Sinapisms to legs; purgatives; enema, and blisters to back of neck. |
| 11 | Recovered | Lived | Do. | Fits came on early in first stage; ceased after delivery. | V.S. to ounces xxx. Purgatives. Delivery with forceps. |
| 12 | Recovered | Lived | Highly albuminous s.g. 1015 | Fits occurred in the second stage; only one soon after delivery. | V.S. to ounces xvj. Cold to head; purgatives; blister to back of neck. Delivery with forceps. |

| No. | Name and Residence. | Date of Attack. | Original Attendant. | Consultant. | Whether the fits came on before, during or after labour. | Whether Multipara or Primipara. | Presentation. |
|---|---|---|---|---|---|---|---|
| 13 | Mrs. — Cumberland House, Hotwells | Feb. 1st, 1862. | Mr. Crichton | Dr. Swayne | During | Primip. | Face |
| 14 | Mrs. H., Stapleton Road | Feb. 8th, 1863. | Mr. Mayor | Dr. Swayne | After | Multip. | Twins—Vertex Breech |
| 15 | Mrs. T., Keynsham | Oct. 13th, 1863. | Mr. Hutchins | Dr. Swayne | Before In 7th Month | Primip. æt. 20. | Vertex |
| 16 | Mrs. P., Cotham Park | Dec. 1st, 1865. | Mr. Sheppard | Dr Swayne | During | Primip. | Brow |
| 17 | Mrs. W., Whiteladies Road | Feb. 10th, 1868. | Dr. T. E. Clark | Dr. Swayne | During | Multip. | Vertex |
| 18 | Mrs. — Jacob's Wells | Jan. 8th, 1862. | Mr. Sawer | Dr. Swayne | During | Multip. | Vertex |
| 19 | Mrs. J., West Street, Bristol | Feb. 13th, 1869. | Mr. Willett | Dr. Swayne | Before Early in 7th Month | Primip. æt. 32. | Vertex |
| 20 | Mrs. H., 2 Catherine Place | Mar. 5th, 1870. | Mr. J. R. Lansdown | Dr. Swayne | Before End of 7th Month | Primip. æt. 19. | Not stated |
| 21 | Mrs. H., Sydenham Hill | Oct. 26th, 1870. | Mr. Taylor | Dr. Swayne | During | Primip. | Twins—Vertex Foot |
| 22 | Mrs. R., Bread Street | Mar. 26th, 1871. | Mr. Wine | Dr. Swayne | During | Multip. æt. 25. | Not stated |
| 23 | Mrs. S., Glo'ster Street, Mall | Aug. 28th, 1872. | Mr. Baretti | Dr. Swayne | Before End of 8th Month | Primip. æt. 38. | Vertex |
| 24 | Mrs. C., Bedminster | May 28th, 1874. | Mr. Keall | Dr. Swayne | Before In 8th Month | Multip. | Not stated |

| No. | Result to Mother. | Result to Child. | State of Urine. | Period of Attack and Duration of Convulsions. | Treatment, &c. |
|---|---|---|---|---|---|
| 13 | Recovered | Lived | 60 per cent. of albumen | Three fits before delivery (which was natural); convulsions worse after; they lasted 18 hours. | Before labour cold to head and sinapisms to legs. After V.S. to ounces xvj., calomel gr. v., jalap 1 scruple; blisters to nape of neck. |
| 14 | Recovered | Lived<br>Lived | 30 per cent. of albumen | Fits came on 14 hours after delivery, and lasted about two days. | Tinct. opii m. xl. (before my arrival). V.S. to ounces xiv. Calomel and jalap āā gr. v.; enema terebinth.; cold to head; blister to nape of neck. |
| 15 | Died | Dead for some hours | 50 per cent. of albumen | Convulsions lasted nearly two days; beginning some hours before labour. | V.S. to ounces xiv. Chloroform during fits. Enema terebinth.; cold to head; blister to nape of neck. |
| 16 | Recovered | Dead | One-sixth albumen | Fits began with second stage; worse after delivery; better after bleeding. | Cold to head, calomel gr. v., enema terebinth. c. ol. ricini (before I arrived). V.S. to ounces xij. (after delivery); blister and sinapisms. Delivery by turning. |
| 17 | Died on third day | Dead | Very albuminous | Convulsions came on early in first stage; ceased after delivery. | Seven leeches to temples; chloroform during fits; dilatation of os uteri. She died from suppression of urine. |
| 18 | Died same night | Dead | Not stated | Convulsions came on early in second stage; none after delivery. | V.S. to ounces xv. Cold to head; purgatives. She died some hours after delivery. |
| 19 | Recovered | Dead | Nearly solid from albumen | Anasarca one month before fits, which occurred in 7th month and lasted about 8 hours. Only one after last V.S. | Purge of calomel and jalap, sinapisms and diuretics (before my arrival). V.S. to ounces xvj. After seven hours V.S. to ounces xvj., blisters to loins, chloroform. Delivery naturally on Feb. 16th. |
| 20 | Died next day | Dead | Not stated | There were 26 fits in 15 hours; none after V.S. | V.S. to ounces xiv. Cold to head; turpentine enemata, sinapisms. Delivery not attempted, as there was no sign of labour. |
| 21 | Recovered | Lived<br>Lived | Albuminuria 3 weeks before attack | Fits began early in labour, and went on for 12 hours; there were none after chloral enema. | Purge of calomel and jalap, and sinapisms (before I arrived). V.S. to ounces xiv., followed by enema of chloral gr. xxx. Some *post partum* hem.rrhage. |
| 22 | Recovered | Dead | Almost solid from albumen | Fits began with first stage—about one every hour; worse and more numerous after. Lasted three days. | Calomel gr. iij. (before my arrival); enema of chloral gr. xxx. V.S. to ounces xij., March 27th. Chloral 1 drachm in two doses. |
| 23 | Died on 20th May (No P.M.E.) | Dead | Very albuminous | Thirty fits before I saw her. No signs of labour. Forty after; lasted two days. Aphasia and dysphagia. | Turpentine enema, and sinapisms (before my arrival) V.S. to ounces xviij. Delivery by podalic version. Chloroform for some hours after. |
| 24 | Recovered | Dead | Very albuminous | Only two convulsions early in the labour. | Cold to head, warmth to feet, sinapisms; enema of turpentine and castor oil. |

| No. | Name and Residence. | Date of Attack. | Original Attendant. | Consultant. | Whether fits came on before, during or after labour. | Whether Mutlipara or Primipara. | Presentation. |
|---|---|---|---|---|---|---|---|
| 25 | Mrs. W., Stanley Villa, West Park | 5 a.m., June 17th, 1874. | Dr. Swayne | Mr. Leonard | Before In 9th Month | Multip. æt. 25. | Vertex |
| 26 | Mrs. C., York Road, Montpelier | 4 a.m., Feb. 10th, 1875. | Mr. Phelps | Dr. Swayne | Before In 9th Month | Primip. æt. 27. | Vertex |
| 27 | Mrs. B., Queen's Parade | Sept., 1876. | Mr. Lansdown | Dr. Swayne | Before In 7th and 8th Months | Primip. æt. 28. | Vertex |
| 28 | Mrs. M., Stapleton Road | 10 p.m., Sept. 30th, 1877. | Mr. Stevens | Dr. Swayne | Before In 9th Month | Primip. | Vertex |
| 29 | Mrs. R., Campbell Street, City Road | 2 a.m., Oct. 6th, 1877. | Mr. Challacombe | Dr. Swayne | Before In 9th Month | Primip. æt. 18. | Vertex |
| 30 | Mrs. L., Southampton Buildings | Dec. 9th, 1877. | Mr. Baretti | Dr. Swayne | During Very early | Multip. | Vertex |
| 31 | Mrs. R., 33 Fernbank Road | April 17th, 1880. | Mr. Lansdown | Dr. Swayne | During | Multip. | Face |
| 32 | Mrs. D., 28 Hampton Park | Sep. 19th, 1880. | Mr. S. H. Swayne | Dr. Swayne | During Just before delivery | Primip. | Vertex |
| 33 | Mrs. M., Berkeley | Nov. 8th, 1880. | Mr. Bridgeman | Dr. Swayne | After About 8 hours after delivery | Multip. | Not stated |
| 34 | Mrs. G., 17 Upper Belgrave Road | Nov. 12th, 1884. | Dr. Swayne | None | After About 2 hours after delivery | Primip. | Vertex |
| 35 | Mrs. B., Barton Hill | June 27th, 1888. | Mr. Baxter | Dr. Swayne | During | Multip. | Not stated |
| 36 | Mrs. H., Weston-s.-Mare | 9 a.m., Aug. 20th, 1890. | Mr. Rossiter | Dr. Fraser Dr. Swayne | After 26 hours after labour | Primip. æt. 37. | Not stated |

| No. | Result to Mother. | Result to Child. | State of Urine. | Period of Attack and Duration of Convulsions. | Treatment, &c. |
|---|---|---|---|---|---|
| 25 | Died twelve hours after attack | Died | Slightly albuminous | No sign of labour before convulsions, which lasted 12 hours. | Purgatives, both draughts and enema. Chloral gr. xxx. Cessation of fits for thr[illegible] hours. Chloral repeated. Os uteri t[illegible] rigid for turning. Delivery by cranioton[illegible] |
| 26 | Recovered | Dead for some hours | Half solid from albumen | Fits lasted from 4 a.m. to 5 p.m.; there were 18 in 13 hours. | Free purging, cold to head, and sinapisı[illegible] (before my arrival). V.S. to ounces x[illegible] Kept under chloroform for 7 hours. N[illegible] more fits. Rigid os notched. Delive[illegible] by forceps. |
| 27 | Recovered | Lived | Albumen about 50 per cent. | Duration of fits uncertain. | V.S. to ounces xij. No fits after. On O[illegible] 8th scarcely a trace of albumen. Favoı[illegible] able confinement on Nov. 4th. |
| 28 | Recovered | Dead for some hours | Very albuminous | Duration of fits about 30 hours. | Chloral gr. xxx. and potass. bromid. 1 dr[illegible] 4tis horis (before my arrival). V.S. [illegible] ounces xxx., with great benefit. D[illegible] livery with forceps. |
| 29 | Died on third day | Lived | Two-thirds albumen | Attacks began before labour, and lasted about 36 hours. | Chloroform inhalation (before I arrive[illegible] Hydrate of chloral gr. xl. by enema; blist[illegible] to nape of neck. Delivery by sh[illegible] forceps. |
| 30 | Recovered | Lived | Nearly half albumen | Fits went on for nearly three days. | Two 1-scruple doses of bromid. potass. (b[illegible] fore I arr ved). Delivery natural. Aft[illegible] delivery, calomel gr. iij., black draug[illegible] and enema terebinth. Cold to hea[illegible] Enema of chloral gr. xxx. |
| 31 | Died soon after delivery | Dead | Albumen about five-sixths of whole | Fits lasted about 8 hours. | Tinct. opii m. xv., and V.S. to ounces [illegible] (before I arrived). Blisters behind ea[illegible] and sinapisms to calves. |
| 32 | Died on second day | Lived | Highly albuminous | Duration of fits uncertain. | V.S. to ounces v. (before I arrived), al[illegible] chloroform. Enema of chloral gr. x[illegible] enema of turpentine and castor oil. |
| 33 | Recovered | Lived | Very albuminous | Fits lasted 15 hours. | V.S. to ounces xvj. Had taken twice g[illegible] xxx. of chloral and gr. xv. of bromide b[illegible] fore I arrived. To continue bromid[illegible] Enema terebinth.; blister to neck. |
| 34 | Recovered | Lived | Albumen about one-third | Only one convulsion, 2 hours after delivery. | Chloral hyd. 1 dr. and potass. bromid. gr. x[illegible] |
| 35 | Died of measles a fortnight after delivery | Dead | Highly albuminous | Fits lasted 11 hours; there were six during labour. | Cold to head; sinapisms to calves of leg[illegible] enema. |
| 36 | Recovered | Lived | Albumen one-fifth | Convulsions lasted 11 hours. | Leeches to temples; 3 oz. of blood thus o[illegible] tained. Calomel and jalap, croton oil [illegible] drop, four doses of bromide (before [illegible] arrived). V.S. to ounces xvj. Chlor[illegible] 1 scruple with bromide gr. xv. |

